# A cross-sectional study on the rate of non-adherence to anti-seizure medications and factors associated with non-adherence among patients with epilepsy

**DOI:** 10.1371/journal.pone.0235674

**Published:** 2020-07-10

**Authors:** Kai Xuan Teh, Nevein Philip Botross Henien, Lyang Shenz Wong, Zoe Kee Hui Wong, Raja Zarina Raja Ismail, Hamdi Najman Achok, Jeevitha Mariapun, Nor’azim Mohd Yunos

**Affiliations:** 1 Clinical School Johor Bahru, Jeffrey Cheah School of Medicine and Health Sciences, Monash University Malaysia, Johor Bahru, Johor, Malaysia; 2 Department of Medicine, Hospital Sultanah Aminah, Johor Bahru, Johor, Malaysia; University of South Australia, AUSTRALIA

## Abstract

**Background:**

Non-adherence to anti-seizure medication (ASM) therapy is an important contributing factor to the higher mortality rate and treatment failure of epilepsy. This study aimed to determine the rate and factors associated with non-adherence to ASM therapy through the WHO five dimensions of medication adherence framework.

**Methods:**

We conducted a cross-sectional study at an outpatient Neurology Clinic of a tertiary government hospital in Malaysia. Between March and July 2019, we identified 217 patients with a confirmed diagnosis of epilepsy, receiving oral ASM therapy and able to administer their medications. We performed a semi-structured interview to gather information on sociodemographic background, clinical and medication history, and perceptions on healthcare services. Adherence to ASM therapy was evaluated using the Medication Compliance Questionnaire (MCQ). Patient’s illness perception was assessed by the Brief Illness Perception Questionnaire (B-IPQ).

**Results:**

208 patients participated in this study. The median age of the study participants was 35 years (IQR 26–44). 58.2% were females and majority, 55.8%, were from the Malay ethnic group. Based on the MCQ scoring, 89 patients (42.8%) were non-adherent. Multiple logistic regression demonstrated that being employed or students (adjusted odds ratio [aOR] 2.26, 95%CI: 1.19–4.29 p = 0.012) and having an average or below average perceived access to pharmacy services (aOR 2.94, 95%CI: 1.38–6.24, p = 0.005) were significant contributors to non-adherence.

**Conclusion:**

Being employed or students and having an average or below average perceived access to pharmacy services were associated with ASM non-adherence Efforts to improve ASM adherence should adopt a comprehensive approach considering the success of adherence is contingent on the interrelationship of multiple dimensions.

## Introduction

Epilepsy is a chronic and disabling neurological disease that affects individuals in all age groups. Approximately 50 million people are affected worldwide and about 80% of patients are found in low and middle-income countries [1, 2]. The median prevalence of lifetime epilepsy in developed countries has been reported as 5.8 per 1,000 population, compared with 15.4 per 1,000 population in developing countries [3]. Globally, about 5 million new cases of epilepsy are diagnosed each year, in developing countries when compared with developed countries (139 versus 49 per 100,000 population) [2].

Epilepsy imposes a significant disease burden and accounts for more than 10 million disability-adjusted life-year (DALY) globally [4, 5]. Patients with epilepsy are at an increased risk of premature mortality than the general population. The explanation for the higher mortality is complex and multifactorial [6], extending beyond the nature of epilepsy and associated comorbidities. Complications of epilepsy range from status epilepticus and sudden unexplained death in epilepsy (SUDEP) to various injuries sustained during an epileptic attack like motor vehicle accidents, falls, burns or drowning. Patients with epilepsy are more likely to suffer from psychiatric comorbidities such as anxiety or depression [7]. The disease is progressive with studies showing gradual neuronal loss and brain atrophy, affecting cognition and memory, after repeated episodes of seizures [8–12]. Anti-seizure medication (ASM) therapy is generally considered as the mainstay treatment for epilepsy with reported 60–70% effectiveness in seizure control [13, 14]. However, 75 to 90% of epilepsy patients have inadequate treatment for their condition, especially in low and middle-income countries [1, 15]. Such high incidence necessitates a better understanding of the crucial element of ASM adherence, an important modifiable factor, in these countries.

Adherence by definition is “the extent to which a person’s behaviour–taking medication, following a diet and/or executing lifestyle changes, corresponds with agreed recommendations from a healthcare provider” [16]. On the other hand, non-adherence can generally be defined as any deviation from the recommended timings or dosages of a prescribed treatment regimen [6]. Non-adherence, whether intentional or unintentional, includes forgetfulness in taking medication, taking more or less than that prescribed or at incorrect timing, premature discontinuation, and failure to refile prescription in pharmacy [17, 18]. The World Health Organization (WHO) proposes five dimensions of factors influencing adherence to long-term therapy and medications: social/economic factors, therapy-related factors, patient-related factors, condition-related factors and healthcare system/healthcare team (HCT)-factors [16].

The prevalence of non-adherence to ASM therapy varies widely in different regions, ranging from 26% to as high as 79% [6, 19]. Objective methods in assessing adherence include pill counts, electronic drug monitoring system, rate of prescription refills, directly observed therapy and monitoring of drug concentrations in body fluids. Subjective methods include patient self-reporting and patient kept-diary [6, 20, 21]. There is no “gold-standard” test for measuring adherence [16] and the most commonly used method is patient self-reporting [6, 19] through the administration of a questionnaire or structured interview. There have been several studies in middle-income countries like Malaysia that evaluated the prevalence and factors associated with ASM non-adherence. However, only a few addressed the WHO healthcare system dimension, particularly the access to pharmacy services. To address the patient-related dimension, we explored the patients’ illness perception, which is thought to be a modifiable risk factor found to be significantly associated with medication adherence in other chronic diseases like diabetes, atrial fibrillation and chronic pulmonary diseases [22–24].

The primary objective of this study was to determine the rate of ASM non-adherence among epilepsy patients. The secondary objective was to explore the factors associated with non-adherence by addressing all five dimensions of the WHO framework of adherence [16].

## Methods

### Study design and selection of participants

We conducted an observational, cross-sectional study at the outpatient Neurology Clinic in Hospital Sultanah Aminah Johor Bahru (HSAJB), the largest tertiary referral centre in southern Malaysia. Between March and July 2019, we screened epilepsy patients under the clinic follow-ups. We included patients aged 18 and above, with a confirmed diagnosis of epilepsy on oral ASM therapy, and who were able to administer their medications. Patients with documented psychiatric disorders, mental disabilities or psychogenic non-epileptic seizures were excluded from the study. Each patient was briefed on the project and a copy of the patient information sheet in both the English and Malay language was provided. Written consent was obtained from all participants.

We used the convenient sampling method, inviting patients who fulfilled the eligibility criteria to participate in the study while waiting for or after their clinic appointments. Using the Raosoft^®^ sample size calculator, we selected a 5% margin of error, a 90% confidence interval, a population size of 20,000 and a response distribution of 64.1% based on findings from a previous study [25]. The calculated sample size was 246 patients.

The study was approved by the Medical Research and Ethics Committee (MREC), Ministry of Health Malaysia (NMRR-18-3423-45385) and the Monash University Human Research Ethics Committee (MUHREC) (Project ID: 19195).

### Assessment of ASM adherence

We evaluated the ASM adherence level using the Medication Compliance Questionnaire (MCQ), developed from two different questionnaires: the Morisky Medication Adherence Scale for epilepsy [26] and the Hill-Bone Compliance to High Blood Pressure Therapy Scale [27]. We chose MCQ as the questionnaire tool as it was available in both English and Malay and used vocabulary that would be better understood by the local Malaysian population. The MCQ has been validated in previous studies, demonstrating an acceptable Cronbach’s alpha value of 0.78 [28, 29]. There are 7 questions in total exploring patient’s adherence behaviour in the MCQ. Each question has a 4-point Likert scale. Scores of 26 or lower are considered non-adherent. Scores of 27 or 28 (1-point subtracted from any one of the unintentional non-adherence questions, i.e. question 1 or question 6) are classified as adherent [28, 29]. Permission to use the questionnaire was granted by the relevant authors.

### Assessment of illness perception

We assessed illness perception using the Brief Illness Perception Questionnaire (B-IPQ), a rapid assessment tool that measures eight different aspects of illness perception: consequences, timeline, personal control, treatment control, identity, concerns, understanding, and emotional representation [30]. A good test-retest reliability and concurrent validity of the original B-IPQ had been demonstrated before [30]. The questionnaire had also been translated to the Malay language with a reported Cronbach’s alpha of 0.65, and a moderate level of psychometric properties, construct validity and test-retest reliability [31, 32]. The B-IPQ is a 9-item instrument that measures illness perception from eight different aspects using an 11-point Likert scale. A higher score reflects a more threatening view of the illness, while a lower score indicates a benign view of the illness [30, 31]. We had obtained permission from the relevant authors to use the B-IPQ in both languages.

### Data collection

We collected sociodemographic information consisting of age, gender, ethnicity, education level, employment status and combined household monthly income. The clinical information recorded comprised of disease-related factors such as the duration of epilepsy, the frequency of epileptic attack in the past one month and the type of epilepsy onset. The therapy-related factors included questions on the number of ASM received, the frequency of ASM taken per day and any history of side effects after taking the ASM. Patient’s illness perception was assessed by the B-IPQ above, while questions assessing their behaviour included the use of any aid(s) for improving adherence and any declining compliance between clinic visits. We explored the perceptions of healthcare system-related factors by assessing the patient’s perception of the effectiveness of ASM, the doctor’s communication skills and the ease of access to pharmacy services.

### Statistical analyses

All statistical analyses were conducted using the statistical software package IBM SPSS Statistics version 24.0 (IBM Corp., Armonk, NY, USA). We compared the differences in socioeconomic background, clinical and medication information, patient’s illness perception and behaviour, and perception towards healthcare systems between the adherent and non-adherent participants. Categorical variables were expressed as frequencies and percentages, normally distributed continuous variables as mean and standard deviation (SD) and non-normally distributed continuous variables as the median and interquartile range (IQR).

We performed univariate logistic regression to determine the various factors associated with non-adherence. Factors with p<0.25 in the univariate analysis were subsequently analysed using multiple logistic regression to adjust for potential confounding effects and identify independent factors associated with non-adherence. A p-value of less than 0.05 was considered statistically significant. The ROC curve, Hosmer-Lemeshow test, classification table and Nagelkerke R-squared were used as measures of goodness of fit for the logistic regression models.

## Results

### Sociodemographic and clinical characteristics

Between March and July 2019, 217 patients were eligible to participate in the study. Nine patients (4.1%) declined to participate in the study and the remaining 208 patients (95.9%) agreed and completed the interview and questionnaires administered.

The patients’ sociodemographic and clinical characteristics are presented in **Table 1**. The median age of our study population was 35 years (IQR, 26–44 years). 58.2% of the patients were female and more than half (55.8%) of the participants were Malay, 28.8% Chinese and 14.9% Indian. 189 participants (90.9%) had achieved secondary or higher education levels and 109 (52.4%) of the study population were employed as full-time or part-time workers or students. The majority (76.9%) had combined household monthly income of less than MYR 5,000. The median duration of epilepsy was 13 years (IQR 5–21). The majority (70.7%) were classified as having focal epilepsy and there was no significant difference between the proportion of patients receiving monotherapy (only one ASM) and polytherapy (2 or more AEDs) (100 patients [48.1%] vs. 108 patients [51.9%]).

**Table 1:** Sociodemographic and clinical characteristics of the patient population (n = 208).

**Sociodemographic characteristics**	
Age in years, median (IQR)	35 (26–44)
Gender, n (%)	
Male	87 (41.8)
Female	121 (58.2)
Ethnicity, n (%)	
Malay	116 (55.8)
Chinese	60 (28.8)
Indian	31 (14.9)
East Malaysian	1 (0.5)
Education level, n (%)	
Primary or lower	19 (9.1)
Secondary or higher	189 (90.9)
Employment status, n (%)	
Employed or student	109 (52.4)
Unemployed, pensioners and housewives	99 (47.6)
Household monthly income, n (%)	
MYR 5,000 or more	48 (23.1)
Less than MYR 5,000	160 (76.9)
**Clinical characteristics**	
Duration of epilepsy in years, median (IQR)	13 (5–21)
Type of epilepsy onset, n (%)	
Focal	147 (70.7)
Generalised	42 (20.2)
Unclassified	19 (9.1)
ASM regimen received, n (%)	
Monotherapy	100 (48.1)
Polytherapy	108 (51.9)

ASM, anti-seizure medication; IQR, interquartile range; MYR, Malaysian Ringgit.

### Assessment of medication adherence

The responses to each question in the MCQ are summarised in **Table 2**. The median overall score of the MCQ was 27 (IQR, 25–28). The scoring for question 1 was the lowest with a mean score of 3.44 (SD 0.69).

**Table 2:** Medication Compliance Questionnaire (MCQ) scoring of the patient population.

Questions	Mean (SD)
1. How often do you forget to take your medicine?	3.44 (0.69)
2. How often do you decide not to take your medicine?	3.79 (0.55)
3. How often do you miss taking your medicine because you feel better?	3.79 (0.54)
4. How often do you decide to take less of your medicine?	3.70 (0.64)
5. How often do you stop taking your medicine because you feel sick due to the effects of the medicine?	3.91 (0.37)
6. How often do you forget to bring along your medicine when you travel away from home?	3.75 (0.55)
7. How often do you not take your medicine because you run out of them at home?	3.84 (0.45)
Mean total score	26.23 (2.39)

SD, standard deviation.

Based on the MCQ scoring system, 89 patients (42.8%) were classified as non-adherent while 119 patients (57.2%) were adherent to their ASM regime. The frequency distribution of their adherence status is shown in **Table 3**.

**Table 3:** Medication Compliance Questionnaire (MCQ) score and adherence status.

Adherence score	n (%)	Status
28 (full score)	82 (39.4)	Adherent
27 (1-point subtracted from either Q1 or Q6)	37 (17.8)	Adherent
27 (1-point subtracted from other questions)	9 (4.3)	Non-adherent
26 or below	80 (38.5)	Non-adherent
Total	208 (100.0)	

### Factors associated with non-adherence to ASM

**Table 4** displayed various factors that were associated with non-adherence, based on the WHO five dimensions of adherence: socioeconomic factors, disease or illness-related factors, therapy (ASM) related factors, patient-related factors (comprised of illness perception and behaviour) and perception on healthcare service-related factors. Factors significantly associated (p<0.05) with non-adherence in the univariate analysis were: younger age, employed workers or students, and having an average or below average perceived access to pharmacy services. Multiple logistic regression revealed that employment status and the perceived ease of access to pharmacy services were significantly associated with non-adherence (p<0.05). Employed workers or students were observed to be associated with higher odds of non-adherence (adjusted OR [aOR] 2.26, 95% CI 1.19–4.29, p = 0.012), when compared to those unemployed, pensioners and housewives. Patients who perceived of having an average or below-average access to pharmacy services were also at increased odds of non-adherence (aOR 2.94, 95% CI 1.38–6.24, p = 0.005).

**Table 4:** Association between various factors and medication non-adherence.

	Non-adherent (n = 89)	Adherent (n = 119)	Unadjusted OR (95% CI)	p value	*Adjusted OR (95% CI)	p value
**Sociodemographic characteristics**						
Age in years, median (IQR)	33 (25–40)	38 (27–48)	0.97 (0.94–0.99)	0.005	0.98 (0.95–1.01)	0.132
Gender, n (%)						
Male	38 (42.7)	49 (41.2)	1.00			
Female	51 (57.3)	70 (58.8)	0.94 (0.54–1.64)	0.826		
Ethnicity, n (%)						
Malay	55 (61.8)	61 (51.3)	1.00			
Chinese	21 (23.6)	39 (32.8)	0.60 (0.31–1.14)	0.478		
Indian	13 (14.6)	18 (15.1)	0.80 (0.36–1.79)			
Education level, n (%)						
Secondary or higher	83 (93.3)	106 (89.1)	1.00			
Primary or lower	6 (6.7)	13 (10.9)	0.59 (0.22–1.62)	0.305		
Employment status, n (%)						
Unemployed, pensioners and housewives	32 (36.0)	67 (56.3)	1.00		1.00	
Employed / student	57 (64.0)	52 (43.7)	2.30 (1.31–4.04)	0.004	2.26 (1.19–4.29)	0.012
Household monthly income, n (%)						
MYR 5,000 or more	19 (21.3)	29 (24.4)	1.00			
Less than MYR 5,000	70 (78.7)	90 (75.6)	1.19 (0.62–2.29)	0.609		
**Disease or illness-related factors**						
Duration of epilepsy in years, median (IQR)	10 (6–20)	13 (5–24)	0.98 (0.95–1.00)	0.081	0.99 (0.96–1.02)	0.577
Frequency of epileptic attack in the past 1 month, median (IQR)	1 (0–1)	0 (0–2)	1.00 (0.95–1.06)	0.879		
**Therapy (ASM) related factors**						
ASM regimen, n (%)						
Monotherapy	49 (55.1)	51 (42.9)	1.00		1.00	
Polytherapy	40 (44.9)	68 (57.1)	0.61 (0.35–1.07)	0.082	0.50 (0.24–1.03)	0.060
Experienced side or adverse effects after taking ASM, n (%)						
No	64 (71.9)	93 (78.2)	1.00			
Yes	25 (28.1)	26 (21.8)	1.40 (0.74–2.64)	0.302		
Frequency of ASM taken per day, n (%)						
Once-daily	7 (7.9)	5 (4.2)	1.00			
Twice-daily	78 (87.6)	109 (91.6)	0.51 (0.16–1.67)	0.537		
Thrice-daily	4 (4.5)	5 (4.2)	0.57 (0.10–3.27)			
**Patients’ illness perception. Score in median (IQR)**						
Consequences	5.0 (3.0–8.0)	5.0 (3.0–8.0)	1.00 (0.92–1.09)	0.930		
Timeline	5.0 (3.0–8.5)	5.0 (3.0–10.0)	0.99 (0.91–1.08)	0.822		
Personal control	5.0 (3.0–8.0)	6.0 (2.0–8.0)	1.00 (0.92–1.09)	0.943		
Treatment control	7.0 (5.0–9.0)	8.0 (5.0–10.0)	0.93 (0.84–1.03)	0.158	0.94 (0.83–1.08)	0.373
Identity	5.0 (3.0–7.0)	4.0 (2.0–7.0)	1.08 (0.98–1.19)	0.130	1.09 (0.97–1.22)	0.142
Concern	9.0 (5.3–10.0)	10.0 (8.0–10.0)	0.91 (0.82–1.00)	0.050	0.94 (0.84–1.05)	0.248
Understanding	7.0 (5.0–9.8)	8.0 (5.0–10.0)	0.96 (0.88–1.05)	0.383		
Emotional response	6.0 (4.0–9.0)	6.0 (3.0–9.0)	1.01 (0.93–1.10)	0.855		
B-IPQ overall score	41.0 (35.0–47.0)	41.0 (33.0–49.0)	1.01 (0.98–1.03)	0.684		
**Patients’ behaviour**						
Use of any aid(s) for improving adherence						
Yes	8 (9.0)	20 (16.8)	1.00		1.00	
No	81 (91.0)	99 (83.2)	2.05 (0.86–4.89)	0.107	1.65 (0.62–4.40)	0.315
Declining compliance between clinic visits						
No	79 (88.8)	112 (94.1)	1.00		1.00	
Yes	10 (11.2)	7 (5.9)	2.03 (0.74–5.55)	0.170	1.95 (0.64–5.96)	0.242
**Perception on healthcare service-related factors**						
Effectiveness of ASM						
Effective	56 (62.9)	89 (74.8)	1.00		1.00	
Neutral	25 (28.1)	27 (22.7)	1.47 (0.78–2.79)	0.076	1.05 (0.50–2.21)	0.286
Ineffective	8 (9.0)	3 (2.5)	4.24 (1.08–16.65)		3.34 (0.74–14.98)	
Assessment of doctor’s communication skills						
Good	52 (58.4)	75 (63.0)	1.00			
Average or below	37 (41.6)	44 (37.0)	1.21 (0.69–2.13)	0.501		
Ease of access to pharmacy services						
Good	59 (66.3)	102 (85.7)	1.00		1.00	
Average or below	30 (33.7)	17 (14.3)	3.05 (1.55–6.00)	0.001	2.94 (1.38–6.24)	0.005

Hosmer-Lemeshow test (p = 0.379), classification table (overall correctly classified = 70.0%), Nagelkerke R square (24.4%) and area under ROC curve (74.6%), was applied to test the model fitness.

*Model has been adjusted for age, employment status, duration of epilepsy, ASM regimen received, treatment control, identity, concern, use of any aid(s) for improving adherence, declining compliance between clinic visits, the effectiveness of ASM and perceived ease of access to pharmacy services

## Discussion

In a population of patients attending follow-ups at the HSAJB outpatient Neurology Clinic the rate of ASM non-adherence between March and July 2019 was found to be 42.8%. This is lower than other local studies where different self-reporting questionnaires were used [25, 33–35]. However, our result was consistent with findings from WHO and two review articles that reported that the ASM non-adherence rate is between 20–80% [6, 16, 19]. Our study also identified factors in the social/economic and health care system facets to be independently associated with non-adherence. The main reason for unintentional medication non-adherence as highlighted by our patients was forgetfulness. This is consistent with findings of cross-sectional studies done in the United States and Brazil which also reported patients’ forgetfulness as the primary contributor of non-adherence [36, 37].

Concerning socioeconomic factors, we found that patients who were either working or students were less likely to adhere to their treatment regimen compared to those who were unemployed, pensioners and housewives. This may be justified by working or academic schedules that preoccupy patients from following their prescribed regimen. Another possible explanation is side effects, particularly drowsiness, which is a commonly reported side effect of ASM [38]. As drowsiness could affect job or academic performance, patients may be tempted to skip doses. Such patients may benefit through the services of the Medication Therapy Adherence Clinic (MTAC) in Malaysia, which is an ambulatory care service that aims to inform and guide patients on their medication usage for better control of their disease [39]. Our findings, however, differ from previous reviews which reported either no association between employment status and adherence [40] or that unemployment was more likely to be associated with poor adherence [6]. One reason for this disparity in findings could be attributed to differences between the classification of employment in our study and other studies. Other socio-economic factors such as gender, ethnicity, education level and monthly income were not found to influence the ASM adherence among our study participants.

Having an average or below-average perceived access to pharmacy services was a significant contributing factor of non-adherence among our study participants. Some common reasons given were the long waiting time and congestion at the outpatient pharmacy department, lack of parking space and also the long travel distance to the hospital. These findings mirror the 2003 WHO report that revealed the negative effect of a long distance from treatment on adherence [16]. Currently, the Malaysian Pharmaceutical Services Division offers several services to improve patients’ access to their monthly medication supply, collectively known as the pharmacy Value Added Services (VAS) [41]. These include the Integrated Drug Dispensing System (IDDS), where patients can choose to collect their monthly ASM supply from the nearest government healthcare facilities (i.e. health clinics) to home and the alternative service of getting patients’ monthly medication supply to be delivered straight to the location of their choice, with minimal delivery fee charges [42]. However, the adoption rate of the VAS is still low [43].and this could be due to the lack of awareness towards pharmacy VAS by patients.

The study did not find significant results pertaining to the patient, therapy or disease-related facets. Patient-related factors such as treatment control, concern, understanding and identity were not found to be statistically different. However, previous studies have shown that patient understanding and perceived lack of benefit of ASM therapy were significant predictors of non-adherence [6, 19, 37]. This contradiction is possibly attributed to the education level of our patients and the high scores in patients’ illness perception which is similar in both the adherent and non-adherent groups in our study. This could be a result of the doctors’ communication skills that were perceived to be good in both groups. We also did not find significant associations between patients’ behaviour and adherence status. A higher proportion of patients in the adherent group reported using memory aids such as pill organisers or alarm reminders as effective measures in ensuring medication adherence. Memory aids have been proposed by the WHO in its 2003 report as a self-management intervention to improve medication adherence [16]. A greater proportion of those in the non-adherent group admitted that they had declining compliance in between clinic visits. This issue is commonly referred to as the white coat adherence, which indicates improved adherence to treatment prior to clinic visits [20, 44]. Suggestive interventions to ameliorate this concern include having frequent follow-up appointments or clinic contacts with patients to improve medication adherence [6, 16]. We did not find significant associations with medication adherence concerning disease-related factors, namely the duration of epilepsy and frequency of attacks. This was in contrast to what was observed in local studies which revealed that a shorter duration of epilepsy and increased seizure frequency were significantly associated with poor ASM adherence [25, 34]. We did, however, observe a trend towards a shorter duration of epilepsy in our non-adherent group. Similar to the patient and disease-related dimensions, we did not find statistically significant associations with adherence for any of the therapy-related factors. This differs from other studies which highlighted significant associations between complicated treatment regimen and ASM adverse effects with poor medication adherence [6, 19, 25, 33]. A possible reason for the discrepancies in our findings may be attributed to our inadequate sample size.

## Limitations

One of the limitations of the study is the use of self-reporting which is subjected to recall bias and the possibility of misinterpretation of questions. We made an effort to minimise such biases by cross-checking information from the patients’ medical records and assisting patients requiring further explanation throughout the interview sessions. Although direct observation method such as measurement of the drugs or its metabolite concentration in the blood may be more accurate in measuring adherence, this method is invasive, very expensive and does not capture the psychometric aspect of nonadherence such as patient’s understanding, believes and attitude. Furthermore, factors such as drugs interaction, physiological and metabolite variations, can also affect the measurement. Future research could employ combinations of methods to measure adherence. Lastly, besides only being a single-centre study, due to time constraints, we were unable to achieve the intended sample size. As a consequence, this study is underpowered. Contradictions between the findings of this study and other studies regarding the factors found to be associated with ASM non-adherence may be partly attributed to the lack of power in detecting statistically significant associations. Despite its limitations, this study has value in providing methodological information on feasibility, especially with regard to study design, patient recruitment and survey questionnaires used to collect information. The results here also serve as preliminary findings for future research on ASM adherence.

## Conclusion

This study serves to inform future studies aiming to provide a greater understanding of the factors that are associated with ASM non-adherence in Malaysia and other countries with similar economies and health systems. Being employed or students and having an average or below average perceived access to pharmacy services seemed to be associated with ASM non-adherence. More research in the country is needed to identify major factors that negatively impact ASM non-adherence. In the perspective of the WHO 5-dimensions of adherence, future strategy formulations aiming to improve ASM adherence should adopt a comprehensive approach considering the success of adherence is contingent on the interrelationship of multiple dimensions.

## Supporting information

S1 File (DOCX)

S2 File (DOCX)
