# Supplementary material for: A cross-sectional study on the rate of non-adherence to anti-seizure medications and factors associated with non-adherence among patients with epilepsy
Source: PLoS One. 2020 Jul 10;15(7):e0235674. doi: 10.1371/journal.pone.0235674 (PMC7351198; doi:10.1371/journal.pone.0235674)
Supplement: S1 File — (DOCX) [file pone.0235674.s001.docx]

**PATIENT INFORMATION SHEET**

**Title of Study:** A cross-sectional study on the prevalence of non-adherence to antiepileptic drugs and factors associated with non-adherence among epilepsy patients in Hospital Sultanah Aminah, Johor Bahru (HSAJB).

Study Site: Neurology Clinic, Hospital Sultanah Aminah Johor Bahru

**Introduction:** This is an invitation for you to participate in our research study. Please take your time to read through and consider this information carefully before you decide if you are willing to participate. Ask the study staff if anything is unclear or if you like more information. After you are properly satisfied that you understand this study, and that you wish to participate, you must sign the informed consent form. Your participation in this study is voluntary. You may withdraw from the study at any time. Your refusal to participate or withdrawal will not affect any medical or health benefits to which you are otherwise entitled. This study have been approved by the Medical Research and Ethics Committee, Ministry of Health Malaysia. An estimated number of 271 participants will be invited to join the research study. The completion of the questionnaires will take around 20-25 minutes.

**Purpose of Study:** The purpose of this study is to determine the prevalence of non-adherence to antiepileptic drugs in Malaysia and to identify the factors associated with non-adherence. Thus, steps can be taken to improve the adherence rates to antiepileptic drugs for the benefit of patients.

**Study Procedure:** If you agreed to this participate in this study, you will be required to answer two questionnaires. The first questionnaire will assess your level of adherence towards your antiepileptic medications and the second questionnaire will gauge your beliefs about medicines. It is of utmost importance that you answer these questions honestly & completely. You can clarify questions which you find difficult to understand with the doctor or medical students involved in this study.

**Risks, side effects and benefits:** The risk of participating in this study will be minimal as it only involves answering questionnaires. You may decline to answer any of the questions that you are uncomfortable with. There may or may not be any benefits to you, however, the information obtained from this study will help improve management of epilepsy in the future.

**Funding the research:** The study participants will not be reimbursed or paid. This is because the study will be conducted during normal visits to the clinic and does not require any further clinic visits or follow-up.

**Termination of study:** The study doctor may due to concerns for your safety, stop the study or your participation at any time. If the study is stopped early for any reason you will be informed and arrangements made for your future care.

**Handling of information**: All your information obtained in this study will be kept and handled in a confidential manner, in accordance with applicable laws and regulations. When publishing or presenting the study results, your identity will not be revealed. Individuals involved in this study, qualified monitors and auditors and governmental or regulatory authorities may inspect and copy your medical records, where appropriate and necessary. The study findings will not be informed to the study participants. However, you may contact the study doctor if you wish to know about the study findings.

**INFORMED CONSENT FORM**

Title of Study: A cross-sectional study on the prevalence of non-adherence to antiepileptic drugs and factors associated with non-adherence among epilepsy patients in Hospital Sultanah Aminah, Johor Bahru (HSAJB).

By signing below I confirm the following:

- I have been given oral and written information for the above study and have read and understood the information given.
- I have had sufficient time to consider participation in the study and have had the opportunity to ask questions and all my questions have been answered satisfactorily.
- I understand that my participation is voluntary and I can at anytime free withdraw from the study without giving a reason and this will in no way affect my future treatment. I am not taking part in any other research study at this time. I understand the risks and benefits, and I freely give my informed consent to participate under the conditions stated. I understand that I must follow the study doctor’s (investigator’s) instructions related to my participation in the study.
- I understand that study staff, qualified monitors and auditors, the sponsor or its affiliates, and governmental or regulatory authorities, have direct access to my medical record in order to make sure that the study is conducted correctly and the data are recorded correctly. All personal details will be treated as STRICTLY CONFIDENTIAL
- I will receive a copy of this subject information/informed consent form signed and dated to bring home.
- I agree/disagree* for my family doctor to be informed of my participation in this study. *(*delete which is not applicable)*

**Subject:**

| Signature: |  | I/C number: |  |
| --- | --- | --- | --- |
| Name: |  | Date: |  |

**Investigator conducting informed consent:**

| Signature: |  | I/C number: |  |
| --- | --- | --- | --- |
| Name: |  | Date: |  |

**Impartial witness:** *(Required if subject is illiterate and contents of participant information sheet is orally communicated to subject)*

| Signature: |  | I/C number: |  |
| --- | --- | --- | --- |
| Name: |  | Date: |  |
