# Supplementary material for: A cross-sectional study on the rate of non-adherence to anti-seizure medications and factors associated with non-adherence among patients with epilepsy
Source: PLoS One. 2020 Jul 10;15(7):e0235674. doi: 10.1371/journal.pone.0235674 (PMC7351198; doi:10.1371/journal.pone.0235674)
Supplement: S2 File — (DOCX) [file pone.0235674.s002.docx]

**RISALAH MAKLUMAT PESERTA**

**Tajuk penyelidikan:** A cross-sectional study on the prevalence of non-adherence to antiepileptic drugs and factors associated with non-adherence among epilepsy patients in Hospital Sultanah Aminah, Johor Bahru (HSAJB).

Tempat penyelidikan: Klinik Neurologi, Hospital Sultanah Aminah Johor Bahru

**Pengenalan:** Anda telah dijemput untuk menyertai penyelidikan ini. Sila ambil masa yang secukupnya untuk membaca dan mempertimbangkan dengan teliti penerangan yang diberi sebelum anda bersetuju untuk menyertai penyelidikan ini. Jika ada sebarang kemusykilan ataupun maklumat lanjut yang anda ingin tahu, anda boleh bertanya dengan mana-mana kakitangan yang terlibat dalam penyelidikan ini. Setelah anda berpuas hati bahawa anda memahami penyelidikan ini, dan anda berminat untuk turut serta, anda dikehendaki untuk menandatangani Borang Persetujuan atau Keizinan Peserta. Penyertaan anda dalam penyelidikan ini adalah secara sukarela. Anda boleh menarik diri daripada penyelidikan ini pada bila-bila masa sahaja. Jika anda tidak mahu menyertai ataupun menarik diri dari penyelidikan ini, tindakan anda tidak akan menjejaskan segala hak dan keistimewaan perubatan kesihatan yang selayaknya anda terima. Penyelidikan ini telah mendapat kelulusan Jawatankuasa Etika dan Penyelidikan Perubatan, Kementerian Kesihatan Malaysia. Seramai 271 orang akan dijemput untuk menyertai penyelidikan ini. Masa yang dianggarkan untuk menjawab borang soal-selidik tersebut ialah 20-25 minit.

**Tujuan penyelidikan:** Tujuan penyelidikan adalah untuk menentukan kadar pematuhan terhadap ubat anti-epileptik di Malaysia dan mengenal pasti faktor-faktor yang berkaitan dengan tahap pematuhan. Maka, langkah-langkah boleh diambil untuk meningkatkan kadar pematuhan terhadap ubat anti-epileptik untuk manfaat pesakit.

**Procedur penyelidikan:** Sekiranya anda bersetuju untuk menyertai penyelidikan ini, anda diperlukan untuk menjawab dua soal selidik. Soal selidik pertama bertujuan menilai tahap pematuhan terhadap ubat anti-epileptik anda dan soal selidik kedua bertujuan memahami kepercayaan anda tentang ubat. Amat penting anda menjawab kesemua soalan dengan jujur dan lengkap. Anda boleh menanyai doktor atau pelajar perubatan yang terlibat dalam kajian ini sekiranya tidak memahami soalan.

**Risiko, kesan sampingan dan manfaat:** Penyelidikan ini mempunyai risiko yang sangat rendah kerana ia hanya melibatkan peserta menjawab beberapa soalan. Anda berhak untuk menarik diri sekiranya anda tidak selesa untuk menjawab soalan-soalan tersebut. Penyelidikan ini mungkin akan mendatangkan manfaat ataupun langsung tiada memberi apa-apa manfaat kepada anda. Segala maklumat yang diperolehi daripada penyelidikan ini akan dapat membantu dalam penambahbaikan pengurusan penyakit epilepsy pada masa depan.

**Siapakah yang membiayai penyelidikan ini:** Peserta penyelidikan tidak akan menerima bayaran penyelidikan ini kerana ianya hanya dijalankan semasa lawatan klinik biasa dan tidak memerlukan sebarang lawatan susulan.

**Penamatan penyelidikan:** Doktor penyelidikan boleh menamatkan penyelidikan ini ataupun menamatkan penyertaan anda dalam penyelidikan ini pada bila-bila masa , jika ia perlu demi keselamatan anda. Jika penyelidikan ini dihentikan terlebih awal, di atas sebab-sebab tertentu, anda akan dimaklumkan dan rawatan yang bakal anda terima selepas itu akan diuruskan.

**Pengendalian maklumat:** Segala maklumat anda yang diperolehi dalam penyelidikan ini akan disimpan dan dikendalikan secara sulit, bersesuaian dengan peraturan-peraturan dan undang-undang yang berkenaan. Sekiranya hasil penyelidikan ini diterbitkan atau dibentangkan kepada orang ramai, identiti anda tidak akan didedahkan. Pihak-pihak tertentu seperti individu yang terlibat dalam penyelidikan, juruaudit dan jurupantau yang terlatih, pihak berkuasa kerajaan atau undang-undang, boleh memeriksa dan membuat salinan laporan perubatan anda jika berkenaan dan diperlukan. Hasil maklumat yang diperolehi dari penyelidikan ini tidak akan didedahkan kepada peserta penyelidikan. Jika anda ingin mengetahui hasil maklumat yang selanjutnya dari penyelidikan ini, anda boleh menghubungi doktor penyelidikan tersebut.

**BORANG PERSETUJUAN/ KEIZINAN PESERTA**

Tajuk Penyelidikan: A cross-sectional study on the prevalence of non-adherence to antiepileptic drugs and factors associated with non-adherence among epilepsy patients in Hospital Sultanah Aminah, Johor Bahru (HSAJB).

Dengan menandatangani di bawah, saya mengesahkan bahawa :

- Saya telah diberi maklumat tentang penyelidikan di atas secara lisan dan bertulis and saya telah membaca dan memahami segala maklumat yang diberikan dalam risalah ini.
- Saya telah diberikan masa yang secukupnya untuk mempertimbangkan penyertaan saya dalam penyelidikan ini dan telah diberi peluang untuk bertanyakan soalan dan semua persoalan saya telah dijawab dengan sempurna dan memuaskan.
- Saya juga faham bahawa penyertaan saya adalah secara sukarela dan pada bila-bila masa saya bebas menarik diri daripada penyelidikan ini tanpa harus memberi sebarang alasan dan ianya sama sekali tidak akan menjejaskan rawatan perubatan saya pada masa akan datang. Saya tidak mengambil bahagian dalam mana-mana penyelidikan lain pada masa ini. Saya juga memahami tentang risiko dan manfaat penyelidikan ini dan saya secara sukarela memberi persetujuan untuk menyertai penyelidikan ini di bawah syarat-syarat yang telah dinyatakan di atas. Saya faham saya harus mematuhi nasihat dan arahan yang berkaitan dengan penyertaan saya dalam penyelidikan ini daripada doktor penyelidikan (penyelidik) .
- Saya faham bahawa kakitangan penyelidikan, pemantau dan juruaudit terlatih , pihak penaja atau gabungannya, dan pihak berkuasa kerajaan atau undang-undang, mempunyai akses langsung dan boleh menyemak laporan perubatan saya bagi memastikan penyelidikan ini dijalankan dengan betul dan data direkodkan dengan betul. Segala maklumat dan data peribadi akan dianggap sebagai SULIT.
- Saya akan menerima satu salinan ‘Risalah Maklumat Peserta dan Borang Persetujuan atau Keizinan Peserta’ yang telah lengkap dengan tarikh dan tandatangan untuk dibawa pulang ke rumah.
- Saya bersetuju/ tidak bersetuju* untuk doktor yang merawat keluarga saya diberitahu tentang penyertaan saya dalam penyelidikan ini. *(*Potong mana yang tidak berkenaan)*

**Subjek:**

| Tandatangan: |  | Nombor K/P: |  |
| --- | --- | --- | --- |
| Nama: |  | Tarikh: |  |

**Penyelidik yang mengendalikan proses menandatangani borang keizinan:**

| Tandatangan: |  | Nombor K/P: |  |
| --- | --- | --- | --- |
| Nama: |  | Tarikh: |  |

**Saksi tidak-berpihak/adil:** *(Diperlukan; jika subjek adalah buta huruf dan kandungan risalah maklumat peserta disampaikan secara lisan kepada subjek)*

Tandatangan: Nombor

K/P:

Nama: Tarikh:
